# Using noninvasive anthropometric indices to develop and validate a predictive model for metabolic syndrome in Chinese adults: a nationwide study

**DOI:** 10.1186/s12902-022-00948-1

**Published:** 2022-03-03

**Authors:** Qian Xu, Li Wang, Jie Ming, Hongwei Cao, Tao Liu, Xinwen Yu, Yuanyuan Bai, Shengru Liang, Ruofan Hu, Li Wang, Changsheng Chen, Jie Zhou, Qiuhe Ji

**Affiliations:** 1 Department of Endocrinology, Xijing Hospital, Air Force Medical University, Changle West Road No. 169, Xi’an, 710032 Shaanxi China; 2 Department of Health Statistics, School of Preventive Medicine, Air Force Medical University, Changle West Road No. 169, Xi’an, 710032 Shaanxi China

**Keywords:** Prediction model, Receiver operating characteristics curves, Calibration curves, Brier score

## Abstract

**Purpose:**

Metabolic syndrome (Mets) is a pathological condition that includes many abnormal metabolic components and requires a simple detection method for rapid use in a large population. The aim of the study was to develop a diagnostic model for Mets in a Chinese population with noninvasive anthropometric and demographic predictors.

**Patients and methods:**

Least absolute shrinkage and selection operator (LASSO) regression was used to screen predictors. A large sample from the China National Diabetes and Metabolic Disorders Survey (CNDMDS) was used to develop the model with logistic regression, and internal, internal-external and external validation were conducted to evaluate the model performance. A score calculator was developed to display the final model.

**Results:**

We evaluated the discrimination and calibration of the model by receiver operator characteristic (ROC) curves and calibration curve analysis. The area under the ROC curves (AUCs) and the Brier score of the original model were 0.88 and 0.122, respectively. The mean AUCs and the mean Brier score of 10-fold cross validation were 0.879 and 0.122, respectively. The mean AUCs and the mean Brier score of internal–external validation were 0.878 and 0.121, respectively. The AUCs and Brier score of external validation were 0.862 and 0.133, respectively.

**Conclusions:**

The model developed in this study has good discrimination and calibration performance. Its stability was proved by internal validation, external validation and internal-external validation. Then, this model has been displayed by a calculator which can exhibit the specific predictive probability for easy use in Chinese population.

**Supplementary Information:**

The online version contains supplementary material available at 10.1186/s12902-022-00948-1.

## Introduction

Metabolic syndrome (Mets) is a group of complex metabolic disorders, including abdominal fat accumulation, high triglyceride, high cholesterol, hypertension, and hyperglycaemia. Mets is not a specific disease but a cluster of multiple risk factors, an intermediate state between health and disease, whose primary role is to bring attention to the possibility of disease in people with an abnormal metabolism. The main components of Mets are insulin resistance and obesity, especially central obesity. Obesity plays an important role in the occurrence and development of Mets [1–4].

Obesity is increasingly recognized as a serious, worldwide public health problem. According to recent studies, obesity is responsible for population-level deaths worldwide [5, 6]. Various methods have been developed to assess the degree of body obesity. Methods for directly measuring or calculating total body fat content include laboratory underwater weighing [7], dual-energy X-ray absorptiometry (DXA) [8–10], magnetic resonance imaging (MRI) [11, 12], skin calliper measurement, girth measurement, bioelectrical impedance analysis (BIA) [8, 13], and some calculation formulas, such as the Clinica Universidad de Navarra-Body Adiposity Estimator (CUN-BAE) [14]. Scholars have also developed anthropometric indices to describe body fat distribution and body shape in humans. The most commonly used indices include body mass index (BMI), waist circumference (WC), hip circumference (HC), waist to hip ratio (WHR), and waist to height ratio (WHtR). Other novel indices include the Body Roundness Index (BRI) [15], A Body Shape Index (ABSI) [16], Body Adiposity Index (BAI) [17], Conicity Index (C-Index) [18], and Abdominal Volume Index (AVI) [19].

BMI is the most commonly used index to assess obesity. However, recent studies have found that people with normal BMI still have a risk of Mets [20, 21]. Our previous studies have also found that normal weight obesity (NWO) populations have a risk of long-term cardiovascular disease and diabetes [22, 23]. It is obvious that the use of BMI alone to assess the risk of metabolic syndrome is flawed. It cannot accurately reflect the degree of obesity of the human body. Therefore, an accurate diagnostic tool is needed to determine whether a person has Mets to better prevent possible cardiovascular diseases and diabetes in the future.

In previous studies on Chinese populations, WHtR, BRI and AVI have been found to have a good ability to discriminate Mets or its components from BMI [21, 24, 25]. However, their AUCs were generally approximately or under 0.8, which did not take into account calibrations, and the studies did not screen these anthropometric indices and demographic information together to fit a more accurate prediction model.

Therefore, this study aims to use a variable selection technique to screen anthropometric indices, establish a simple metabolic diagnosis model in a Chinese population, evaluate the model performance by discrimination and calibration and assess the overfit by internal, internal-external and external validation. The final model will be displayed by a scoring system in an Excel document [26, 27].

This study is reported in accordance with the TRIPOD (Transparent Reporting of a multivariable prediction model for Individual Prognosis or Diagnosis) Statement, a guideline specifically designed for the reporting of studies developing or validating a multivariable prediction model [28].

## Methods

### Source of data and Participants

The development set for this study came from a large cross-sectional study: the China National Diabetes and Metabolic Disorders Survey (CNDMDS). This is a nationwide epidemiological survey from June 2007 to May 2008, which was completed by 17 clinical centres in 14 provinces and cities across the country. A multistage, stratified cluster sampling method was used to select persons aged 20 years or older. In total, 152 urban street districts and 112 rural villages were selected, 54,240 participants were invited to participate in the study, and 47,325 persons (18,976 men and 28,349 women) accepted the invitation. Finally, 46,239 adults completed the survey. The validation set came from phase 3 follow-up surveys of CNDMDS in Shaanxi Province, which were conducted from October 2016 to November 2017. A total of 1072 participants were included in phase 3 follow-up surveys. Relevant information on the dataset can be found in our previous studies [22, 23, 29, 30].

Because some centres did not conduct BIA testing in the development set, we included 27,494 participants from 9 centres. Because of the measurement error of BIA, any data points of PBF that were 3 interquartile ranges below the first quartile (Q1) or 3 interquartile ranges above the third quartile (Q3) were considered outliers and excluded from the analysis. Some participants with missing information on family history, height, weight, BP (blood pressure), TGs (triglycerides), HDL-C (high-density lipoprotein cholesterol), and BG (blood glucose) were also excluded. Then, participants who were using antihypertensive medications, lipid medications and diabetes medications were excluded.

**Fig. 1:**
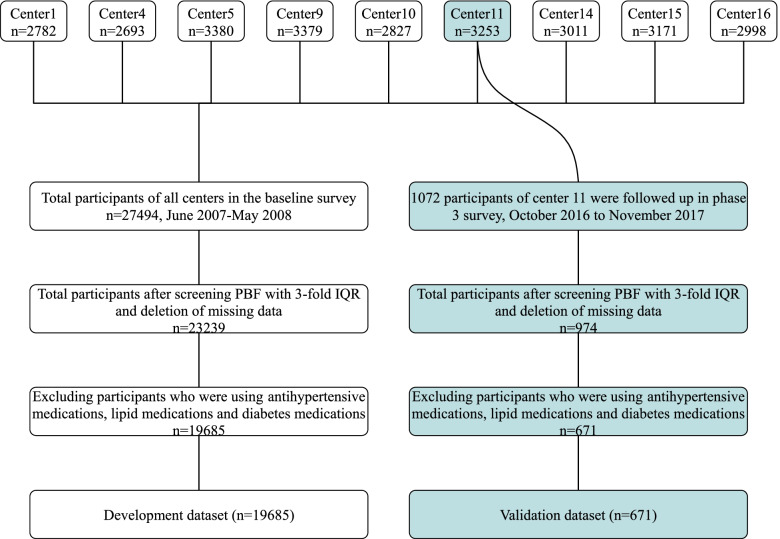
The flowchart of details of participant inclusion and exclusion criteria of development dataset and validation dataset

Finally, a total of 19,685 participants from 9 centres were included in the development set. Similarly, a total of 671 participants were included in the validation set (Fig. 1).

### Outcome

The outcome definition is according to the Asian criteria of the 2009 Joint Interim.

Statement of several international societies (2009 JIS). Specifically, participants having three or more of the following clinical measures were considered as having Mets:

1. central obesity (WC ≥ 90 cm (males) or ≥ 80 cm (females));
2. elevated BP: SBP (systolic blood pressure) ≥130 mmHg or DBP (diastolic blood pressure) ≥85 mmHg, or the use of antihypertensive medications;
3. elevated fasting TG: ≥ 1.69 mmol/l or the use of lipid medications;
4. elevated FBG (fasting blood glucose): ≥ 5.6 mmol/l or the use of diabetes medications;
5. decreased HDL-C: < 1.04 mmol/l (males) or < 1.29 mmol/l (females) [4].

### Predictors

According to a previous review [24, 25, 31–48] and our current study [22, 23, 30], the following variables were considered candidate predictors to judge a patient with or without metabolism: age, sex, education level, smoking history, physical activity history, family history of metabolic disorders (abbreviated as family history), SBP, DBP, WC, HC, WHR, WHtR, BRI, BMI, PBF, ABSI, CUN_BAE, BAI, C-Index, and AVI.

Participants were asked to wear a single layer of light clothing when they were measured for weight, height, PBF, WC and HC. The definition or calculation formula of each factor is as follows:

Age was measured as of the date of completing the survey.

Education level: Those who had a college degree or above were defined as having a high education level, and those who had a secondary education or below were defined as having a low education level.

Smoking history: Those who had smoked more than 100 cigarettes in their lifetime and were still smoking now were defined as category yes, those who had smoked in the past and had quit for more than 1year were defined as category quit, and the others who had never smoked were defined as category no.

Physical activity history: Those who had regular exercise more than three times a week with each session lasting at least half an hour were defined as category regular, and the others were defined as category never.

Family history of metabolic disorders: A family history of hypertension was defined as having at least one of the parents, siblings, and children diagnosed with hypertension in their lifetime. Similarly, we defined the family history of diabetes and hyperlipidaemia and calculated the cumulative number of these three metabolic disorders.

Blood pressure: A mercury column sphygmomanometer was used to measure blood pressure. The participant was required to rest quietly and relax before measurement.

Height and weight: A height and weight scale was used to measure height and weight. The measurement results were required to be accurate to 0.5 cm and 0.5 kg.

WC andHC: A measuring tape was used to measure WC andHC. The measurement results were required to be accurate to 0.5 cm.

PBF: A Tanita body composition analyser (TBF-300 WA; Tanita Corporation Limited, Tokyo, Japan) was used to measure PBF.

Calculation formula:

$$WHR= WC(cm)/ HC(cm)$$

$$WHtR= WC(cm)/ height(cm)$$

$$BRI=364.2-365.5\times {\left\{1-{\left[\frac{WC(cm)}{\pi \times height(cm)}\right]}^2\right\}}^{\frac{1}{2}}$$

$$BMI= weight\ (kg)/{height}^2(m)$$

$$ABSI= WC(m)/\left[{BMI}^{\frac{2}{3}}\times {height}^{\frac{1}{2}}(m)\right]$$

$$CUN\_ BAE=-44.988+\left(0.503\times age\right)+\left(10.689\times sex\right)+\left(3.172\times BMI\right)-\left(0.026\times {BMI}^2\right)+\left(0.181\times BMI\times sex\right)-\left(0.02\times BMI\times age\right)-\left(0.005\times {BMI}^2\times sex\right)+\left(0.00021\times {BMI}^2\times age\ \right)$$

*Where male = 0 and female = 1 for Sex.*

$$C- Index= WC(m)/\left[0.019\times \sqrt{weight(kg)/ height(m)}\right]$$

$$AVI=\left\{2\times {WC}^2(cm)+0.7\times {\left[ WC(cm)- HC(cm)\right]}^2\right\}/1000$$

### Missing data and Sample size

All missing values were removed, and a total of 19,685 participants were included in this study. According to relevant research, the events per variable (EPV) required in multivariate analysis must be 10 or greater, namely, the positive event should be more than 10 times the number of predictors. A total of 6505 participants were diagnosed with Mets, so the sample size was large enough [27, 49].

### Statistical analysis

The baseline information in the dataset was compared using the chi-square test or Fisher’s exact test for categorical variables and the two-sample t test or Mann–Whitney U test for continuous variables. Anthropometric indices, SBP, DBP and age were analysed as continuous variables, while sex, education level, smoking history, physical activity history and family history were analysed as discrete variables. To avoid overfitting of the prediction model and multicollinearity among predictors, least absolute shrinkage and selection operator (LASSO) regression was performed to screen the predicted variables. With the increase in the penalty parameter lambda, the coefficients of each predictor shrank, and the number of predictors was reduced. Then, according to the number of predictors, area under the receiver operator characteristic curves (AUCs), and misclassification error, we selected some factors as independent variables to establish the logistic regression model [26].

The overall model performance was evaluated mainly by the Brier score, which is simply defined as (*y* − *p*) ^ 2, with *y* the outcome and *p* the prediction for each subject. The average score of all subjects is the Brier score of the model. It refers to the distance between the predicted outcome and actual outcome. The Brier score is a proper scoring rule that combines calibration and discrimination, similar to Nagelkerke’s R^2^ [27]. Then, the discrimination and calibration of the model were evaluated. Harrell’s concordance statistic (C-index) is the most commonly used performance index to measure the discriminative ability of generalized linear regression models. It gives the probability a randomly selected subject which experienced an event had a higher risk score than a subject which had not experienced the event. For a binary outcome, the C statistic was the same as the area under the receiver operating characteristic (ROC) curve (AUC). Calibration refers to the agreement between observed outcomes and predictions, which refers to if we observe *p*% positive event among subjects with a predicted risk of *p*% in ideally situation. Calibration was usually evaluated by a calibration plot with predictions on the x-axis and the outcome on the y-axis, meaning the agreement between observed outcomes and predictions, perfect predictions should be at the 45° line. For different sample size data, different smoothing techniques were used to estimate the difference between the observed probabilities of outcomes and the prediction probabilities. As Steyerberg recommended, locally weighted least squares regression (loess) smoother was used to draw the calibration curve of development set because its sample size is greater than 5000, and restricted cubic spline (RCS) smoother was used to draw the calibration curve of validation set [26, 50].

For internal validation, we computed using a 10-fold cross validation procedure to validate the model performance: 90% of the data were used to train the model, and the remaining 10% were used to compute the model performance; this process was repeated 10 times.

For internal-external validation, as Steyerberg and Harrell advocated [51], 8 of the 9 centres’ data were selected as the development set to generate the model, the data of the 1 remaining centre was used as a validation set to evaluate the model performance, and this process was repeated to verify each centre sequentially and calculate the mean performance.

For external validation, we used a new dataset as a validation cohort to evaluate the model performance of the original model.

The final model is displayed by a scoring system made in Excel, which is convenient to use. All statistical analyses were performed using R 4.1.1 (R Foundation for Statistical Computing, Vienna, Austria) and RStudio 1.4.1106, the Integrated Development for R (250 Northern Ave, Boston, MA 02210, USA). The package “compareGroups” was used to make baseline tables, the package “glmnet” was used to perform LASSO regression, the package “rms” was used to build a prediction model, the package “pROC” was used to plot ROC curves, and the package “CalibrationCurves” was used to plot calibration curves.

## Results

A total of 19,685 participants from 9 centres were included in model training, and 5138 participants were diagnosed with Mets, accounting for 26.1% of the total number. The differences in all baseline characteristics and predictors between the Mets group and the Non-Mets group are shown in Table 1. Categorical variables were compared using the chi-squared or Fisher’s exact test, and all continuous variables were verified to conform to a normal distribution, so they were compared using the t test or ANOVA (analysis of variance) test. The *p* values of the overall groups significance were shown in the table.

**Table 1:** Baseline data of each candidate predictor in the development set

	[ALL]	Non-Mets	Mets	p.overall
	***N*** = 19,685	***N*** = 14,547	***N*** = 5138	
Age	43.0 (13.1)	41.2 (12.9)	48.1 (12.5)	< 0.001
Sex:				0.069
Male	7982 (40.5%)	5843 (40.2%)	2139 (41.6%)	
Female	11,703 (59.5%)	8704 (59.8%)	2999 (58.4%)	
EDU:				< 0.001
Low	15,055 (76.5%)	10,810 (74.3%)	4245 (82.6%)	
High	4630 (23.5%)	3737 (25.7%)	893 (17.4%)	
Smoke:				< 0.001
No	14,791 (75.1%)	11,005 (75.7%)	3786 (73.7%)	
Quit	719 (3.65%)	486 (3.34%)	233 (4.53%)	
Yes	4175 (21.2%)	3056 (21.0%)	1119 (21.8%)	
PhysicalActivity:				0.343
Never	12,668 (64.4%)	9333 (64.2%)	3335 (64.9%)	
Regular	7017 (35.6%)	5214 (35.8%)	1803 (35.1%)	
FamilyHistory:				< 0.001
Non	11,576 (58.8%)	8673 (59.6%)	2903 (56.5%)	
One	5258 (26.7%)	3826 (26.3%)	1432 (27.9%)	
Two	2175 (11.0%)	1578 (10.8%)	597 (11.6%)	
Three	676 (3.43%)	470 (3.23%)	206 (4.01%)	
SBP	121 (17.7)	117 (15.3)	133 (18.0)	0.000
DBP	77.9 (10.6)	75.6 (9.61)	84.3 (10.6)	0.000
WC	81.2 (10.5)	78.2 (9.35)	89.9 (8.75)	0.000
Hip	95.2 (7.85)	93.3 (7.09)	101 (7.40)	0.000
WHR	0.85 (0.07)	0.84 (0.07)	0.89 (0.07)	0.000
WHtR	0.50 (0.06)	0.48 (0.06)	0.55 (0.05)	0.000
BRI	3.46 (1.22)	3.10 (1.04)	4.48 (1.10)	0.000
BMI	24.0 (3.59)	23.0 (3.18)	26.6 (3.32)	0.000
PBF	28.7 (7.87)	27.0 (7.31)	33.5 (7.45)	0.000
ABSI	0.08 (0.01)	0.08 (0.01)	0.08 (0.01)	< 0.001
CUN-BAE	29.2 (7.92)	27.7 (7.62)	33.6 (7.02)	0.000
BAI	51.1 (5.61)	49.7 (5.06)	54.9 (5.33)	0.000
C-Index	1.20 (0.10)	1.18 (0.09)	1.26 (0.08)	0.000
AVI	13.6 (3.45)	12.6 (2.95)	16.4 (3.19)	0.000

### Model selection and development

**Fig. 2:**
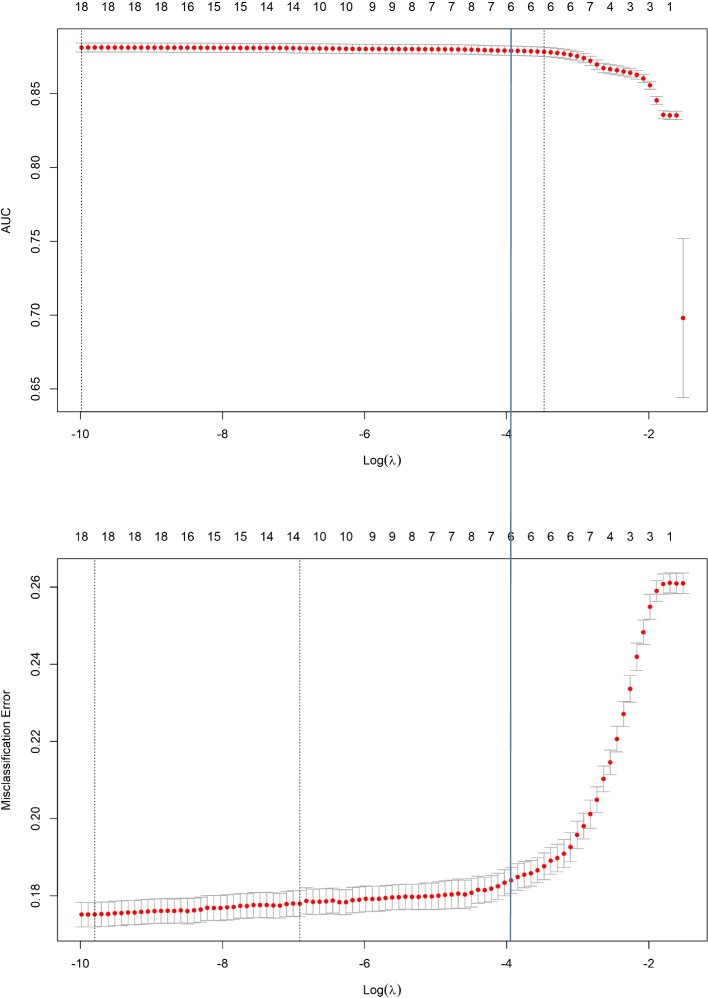
Plot the change trend of AUC and misclassification error with the increase of Log(λ). The AUC and misclassification error on the left side of the blue solid line do not change significantly. The number of predictors corresponding to the blue solid line is 6, and the AUC is above 0.85, the misclassification error is below 0.2

To reduce the overfitting of the model and the collinearity among the dependent variables, the LASSO method was used to screen the prediction factors, which achieves the selection of predictors by shrinking some coefficients to zero by penalizing the absolute values of the regression coefficients (Fig. 2).

Figure 2 shows the change trend of AUCs and misclassification error (percentage of predicted values that do not match observed known values; the lower the misclassification error is, the better the model) with the increase in log (λ). The coefficients for the final model can be chosen at the lowest cross validated log (λ) value (the position of a black dotted line on the left of Fig. 2), or more conservatively, at a 1 standard error larger value of log(λ) (the position of a black dotted line on the right of Fig. 2). However, we conclude that in the first half of the increase in the contraction penalty λ, the increase in misclassification error and the decrease in AUCs are not significant. After comprehensive consideration, we chose the corresponding model at a 1 standard error larger value of log(λ) for AUCs; at this time, the misclassification error was close to 0.2, and the AUCs were still higher than 0.85.

Therefore, the model finally included SBP, DBP, WC, WHtR, PBF and CUN_BAE as predictors, and we used these 6 variables to establish the logistic regression equation as a final model. The regression coefficient of each variable and intercept is shown in our score calculator*.*

### Model performance and validation: discrimination and calibration

**Fig. 3:**
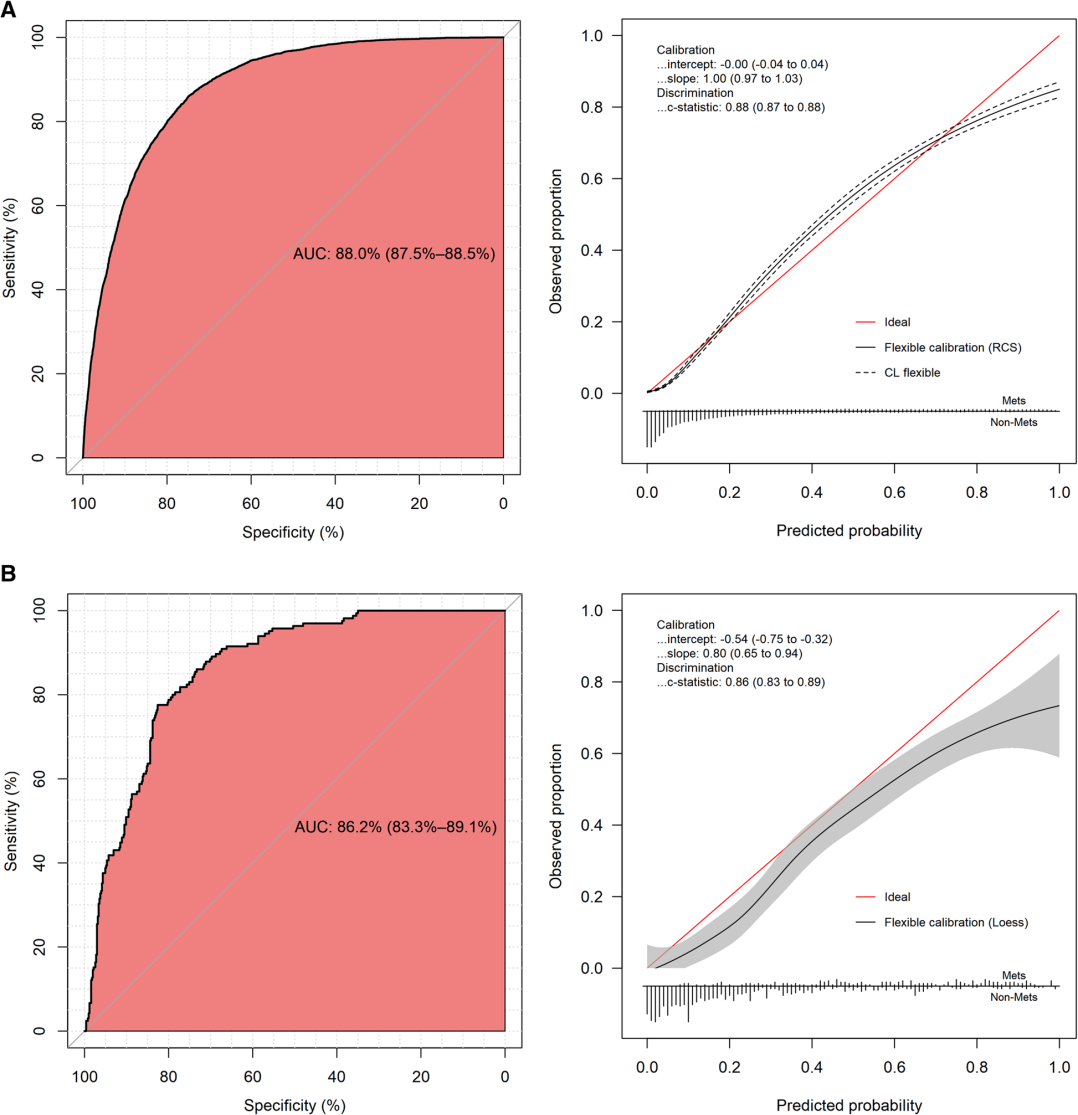
The ROC curves and calibration curves of development set (**A**) and validation set (**B**). In the ROC curve, the y-axis is the sensitivity from 0 to 100%, and the x-axis is the specificity from 100% to 0. The y-axis of the calibration curve is the proportion of positive outcomes observed in the corresponding group, and the x-axis is the average prediction probability of the model, the perfect prediction should be on the 45-degree line. The calibration curve of development set is constructed with the restricted cubic spline (RCS) smoother and the calibration curve of validation set is constructed with the “loess” smoother

Model discrimination was evaluated by ROC curves, and model calibration was evaluated by calibration curves. We assessed discrimination and calibration in the development set through internal (via 10-fold cross validation), internal–external (across centres) and external (via another dataset) methods. The ROC curves and calibration curves are shown in Fig. 3A*.* In the original model performance, the C statistic/AUC was 0.88 (95% CI: 0.875-0.885), and the Brier score was 0.122. After 10-fold cross validation, the mean C statistic/AUCs was 0.879, and the mean Brier score was 0.122 *(*Table 2*)*, which are extremely close to the original model performance. In the internal-external validation, the model performance of each centre is shown in Table 3. The mean C statistic/AUC of the 9 centres was 0.878, and the mean Brier score of the 9 centres was 0.121, which were also very close to the original model performance. In the validation set, the C statistic/AUC was 0.862 (95% CI: 0.833-0.891), and the Brier score was 0.133 (Fig. 3B). Consequently, these results suggested that the prediction models had good performance.

**Table 2:** A total of 10 10-fold cross-validation was carried out. The AUC and Brier Score calculated for each validation are shown in this table, and the average value is calculated at the end

Times	AUC	Brier Score
1	0.881	0.118
2	0.885	0.119
3	0.884	0.123
4	0.869	0.125
5	0.879	0.125
6	0.881	0.117
7	0.889	0.121
8	0.884	0.119
9	0.859	0.132
10	0.879	0.121
Mean	0.879	0.122

**Table 3:** Each center in turn serves as a validation set. The AUC and Brier Score calculated for each validation are shown in this table, and the average value is calculated at the end

Center	N	AUC	Brier Score
1	2135	0.881	0.127
4	2383	0.871	0.125
5	2582	0.895	0.093
9	1755	0.876	0.118
10	1274	0.900	0.097
11	1991	0.884	0.120
14	2344	0.836	0.154
15	2615	0.881	0.134
16	2606	0.882	0.122
Mean		0.878	0.121

### Model visualization: making a risk score calculator

**Fig. 4:**
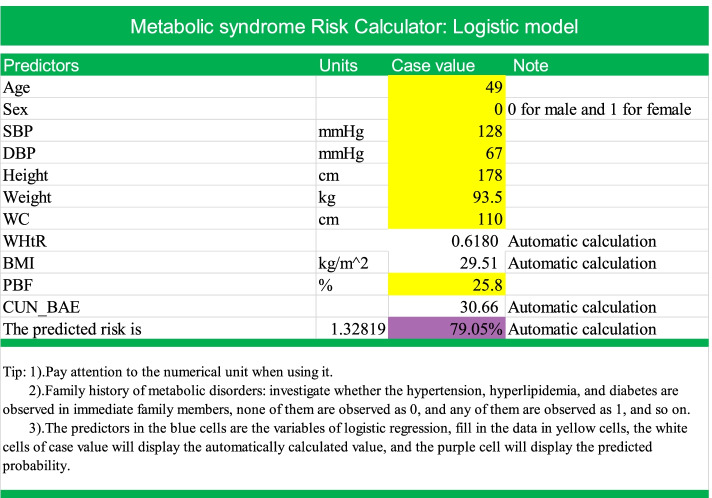
The models were presented as logistic regression equations in this Excel document, it will calculate the predictor (lp) first. According to logit transformation, $\mathrm{lp}=\mathrm{logit}\left(\mathrm{p}\right)=\log \left(\frac{\mathrm{p}}{1-\mathrm{p}}\right)$, so $\mathrm{p}=\frac{\exp \left(\mathrm{lp}\right)}{1+\exp \left(\mathrm{lp}\right)}$, the p value will be displayed in the purple cell. *p=The prediction of probability of Mets.

The formulas of these anthropometric indices were quite complicated, and it is not convenient to calculate all anthropometric indices manually; therefore, we created an Excel file that included all of the formulas and coefficients of predictors. When entering age, height, weight, WC and other simple values, it can automatically calculate anthropometric indices and the probability of Mets (Fig. 4). We randomly entered the data of a participant as a demonstration; this was a 49-year-old man with Mets, and his predicted probability of Mets calculated by the model was 79.05%.

## Discussion

To our knowledge, this is the first study that developed a diagnostic model with noninvasive anthropometric indices for Mets in a large representative Chinese population and many minority groups. When screening predictive variables, we excluded indices such as the triglyceride-glucose index (TyG) [32] and the visceral adiposity index (VAI) because their calculation formulas included serum triglyceride or HDL-C, which were included in the diagnosis standard of Mets, and we also hoped to use some noninvasive measurement indices as predictors.

LASSO regression was used to improve the multicollinearity among predictors and prevent model overfitting, and 6 predictors were selected by shrinking the coefficients towards zero. Among the predictors, SBP, DBP and WC are part of the diagnostic criteria for Mets, and WHtR, PBF and CUN_BAE have been proven to have separate predictive values for Mets in previous studies [24, 41, 52], so the prediction model established by these 6 predictors has very good performance.

To evaluate the performance of the model, we calculated the AUCs, plotted the calibration curve and calculated the Brier score of the model to evaluate the calibration. The AUCs of the model are greater than 0.8, which is considered to indicate better discrimination, and the Brier score of the model is less than 0.2, which is considered to indicate better calibration. However, we also found that the model was significantly overestimated in groups with high predictive probabilities: at the right third of the calibration curve, the actual prevalence was lower than the average predictive probability, and this result is consistent with Wang’s study. The AUCs of this study is slightly lower than that of Wang’s study (AUC 0.901), but the development set of Wang’s study came from Spanish workers without external validation set. And the model of Wang’s study was shown by nomogram, the corresponding indicator such as body fat percentage (calculated as body fat percentage = 1.2 × (BMI) + 0.23 × age (years) − 10.8 × gender (male, 1; female, 0) − 5.4) need to be calculated well in advance before using the nomogram [53]. Similarly, Zhang’s study established a prediction model for 4-year risk of Mets with age, TC (serum total cholesterol), UA (serum uric acid), ALT (Alanine aminotransferase), and BMI, this was a longitudinal study (AUC 0.783, Brier score 0.156) but included invasive biochemical indicators as predictors [54]. Compared to Zhang’s study, our study lacks longitudinal validation because our study is a cross-sectional study, we cannot use this model to predict the probability of being diagnosed with Mets in the future, But we believe that this overestimation is useful to remind people of metabolic disorders. People who have been misdiagnosed have a high predictive probability, and they are likely to have metabolic problems in the future. We will conduct research to verify the ability of this model to predict the long-term outcomes of Mets.

We carried out 10-fold cross internal validation, external validation, and multicentre internal-external validation, which were rarely conducted at the same time in previous studies. The average AUCs and Brier score showed no significant changes in the internal validation and multicentre internal-external validation and were almost equal to the values in the original model. The performance of the validation set dropped slightly but was still in a good range. These results showed that the model has good performance and a stable predictive ability in population of different provinces in China, which is conducive for use in different centres.

Previous studies have mostly used nomographs to display predictive models, but nomographs are not accurate enough and convenient to use, and some predictors in this study need to be calculated indirectly, so a scoring system is used to display the model.

There are still some shortcomings in our study. 1) This study is a national study that was focused on investigating the prevalence of diabetes in China. The lifestyle, exercise history and eating habits of participants were kept simple, resulting in poor predictive ability of relevant predictors, and their coefficient in LASSO regression shrank to zero early; they were not selected as the final prediction factor. 2) The first phase of this study was conducted in approximately 2008. Due to the limited personnel and equipment, some new anthropometric indices could not be recorded. In future research, we will discuss how to add new prediction factors to improve the performance of the model. 3) In the 10-fold cross-validation, the performance of the model is not obviously decreased, but the performance of some centres has declined in internal and external validation, indicating that regional and ethnic differences may adversely affect the model’s performance. 4) The external validation datasets came from Shaanxi Province’s phase 3 follow-up data. Strictly speaking, the validation of these repeated participants belongs to the period validation of external validation, so the model needs more studies to conduct external validation to confirm its stability.

## Conclusions

The model developed in this study has good discrimination and calibration performance, external validation and internal-external validation show that this model can maintain good stability in different space or time. Then, this model has been displayed by a calculator which can easily, accurately and quickly identify individuals with risk of Mets, help clinicians understand the physical condition of patients, and formulate treatment or rehabilitation plans in time.

## Supplementary Information

**Additional file 1.**

## Data Availability

The datasets analysed during the current study are not publicly available because this is a national study covering the intellectual property rights of these 14 centres. However, some data can be obtained from the corresponding author upon reasonable request.
